# Statistical strategies to improve the efficiency of molecular studies of colorectal cancer prognosis

**DOI:** 10.1038/sj.bjc.6604792

**Published:** 2008-11-18

**Authors:** P Qu, H Chu, J G Ibrahim, J Peacock, X J Shen, J Tepper, R S Sandler, T O Keku

**Affiliations:** 1 Cancer Research and Biostatistics (CRAB), 1730 Minor Ave Suite 1900, Seattle, WA 98101, USA; 2 Department of Biostatistics, School of Public Health, University of North Carolina, Chapel Hill, NC 27599, USA; 3 Department of Medicine and Center for Gastrointestinal Biology and Disease, University of North Carolina, Chapel Hill, NC 27599, USA; 4 Department of Radiation Oncology, School of Medicine, University of North Carolina, Chapel Hill, NC 27599, USA

**Keywords:** efficiency, stopping rule, variability, survival, molecular markers

## Abstract

The evaluation of tumour molecular markers may be beneficial in prognosis and predictive in therapy. We develop a stopping rule approach to assist in the efficient utilisation of resources and samples involved in such evaluations. This approach has application in determining whether a specific molecular marker has sufficient variability to yield meaningful results after the evaluation of molecular markers in the first *n* patients in a study of sample size *N* (*n*⩽*N*). We evaluated colorectal tumours for mutations (microsatellite instability, K-ras, B-raf, PI3 kinase, and TGF*β*R-II) by PCR and protein markers (Bcl2, cyclin D1, E-cadherin, hMLH1, ki67, MDM2, and P53) by immunohistochemistry. Using this method, we identified and abandoned potentially uninformative molecular markers in favour of more promising candidates. This approach conserves tissue resources, time, and money, and may be applicable to other studies.

Research on the molecular biology of colorectal cancer has increased our expectation that a better understanding of molecular changes in colorectal tumours may improve our knowledge of aetiology and treatment. Recently, investigators have recognised that molecular characteristics of colorectal cancers are associated with prognosis and therapeutic response. Studies suggest that some of the major genetic players in colorectal neoplasia, such as p53 mutations, are associated with poorer prognosis (Hardingham *et al*, 1998). Other studies report correlations between K-ras mutations, tumour stage, and survival (Andreyev *et al*, 1998; Samowitz *et al*, 2000). In a population-based study of 607 colorectal cancer patients, Gryfe *et al* (2000) observed that high-frequency microsatellite instability (MSI) conferred significant survival advantage independent of other prognostic factors including tumour stage.

Molecular studies in colorectal cancer may help us better understand how genetic alterations could alter prognosis or impact response to cytotoxic agents. However, there are limitations in the analysis of molecular markers in studies of colorectal cancer prognosis. Oftentimes, studies have a limited amount of tissue samples or have samples from a small number of subjects. Furthermore, variation in the expression of markers in tumour samples might be too small to detect differences in prognosis, thus limiting the utility of some markers. Therefore, there is a need to devise strategies to utilise resources efficiently in studies of molecular markers of prognosis.

In the conduct of a population-based study to determine prognostic and predictive molecular factors for colorectal cancer, we used data from more than 100 patients to develop a strategy to determine whether specific molecular markers possess sufficient variability to yield meaningful results in a study of sample size 1000. Using this method, molecular markers that were unlikely to be informative were abandoned in an early stage of the study in favour of mutations or protein markers showing more promise. This method allowed us to conserve time and resources, and may be applicable to other molecular studies.

## Materials and methods

We are conducting a population-based study of colorectal cancer in 33 county areas of North Carolina. This study, Cancer Care Outcomes and Surveillance (CanCORS), is a multicentre population-based study, funded by the National Cancer Institute, to evaluate patient, physician. and treatment factors that influence colorectal cancer outcomes. As part of the CanCORS study at the University of North Carolina, we collected tumour tissue on consenting subjects, and constructed tissue microarrays (Kononen *et al*, 1998) to be used for immunohistochemistry and mutational analysis as part of the UNC GI Specialized Programme in Research Excellence (SPORE) grant. We enrolled 1000 patients (*N*=1000) into the study, and the study was approved by the Institutional review board (IRB) of the UNC School of Medicine. From more than 100 patients, we evaluated genetic mutations in p53 (Angelopoulou and Diamandis, 1998; Curtin *et al*, 2004), K-ras, B-raf, TGF*β*R-II, MSI (Boland *et al*, 1998), and examined protein expression of MDM2, BCl-2, cyclin D1, Ki67, P53, hMLH1, and E-cadherin by immunohistochemistry using commercial antibodies.

### Binary mutation marker data

In our study of *N*=1000 patients, our objective was to develop a stopping rule that might be applied after the first *n* patients were evaluated (*n*⩽*N*) to improve efficiency and lower cost. A binary mutation marker variable takes a value of 0 or 1 to represent the absence or presence of a mutation, respectively. To assess the effectiveness of any marker, one typically employs a regression model to correlate the marker variable with the outcome. A problem arises in the early stages of a study when time-to-event outcomes are not yet available because of short follow-up, hindering the evaluation of marker effectiveness in terms of survival. However, one can still make some informative decisions on marker effectiveness by evaluating marker variability. If among the first *n* (*n*⩽*N*) patients most have either mutations or non-mutations, it suggests that the marker has little variability and likely little impact on prognosis.

To evaluate marker effectiveness through marker variability without survival outcome data, we find it appropriate to use the power and sample size relation. Let *α* denote significance level and *Z*_1−*α*_ the (1−*α*) × 100% percentile from the standard normal distribution. Assuming the Cox proportional hazards model, Schoenfeld (1983) derived a sample size and power relation for two sample comparisons (eg, mutations *vs* non-mutations) in which the proportion of the mutation group *p* satisfies: 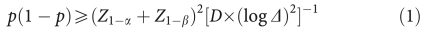 where *Δ* is the hazard ratio between two samples, *D* is the total deaths among *N* patients (which can be also written as *D*=N^*^d where *d* is the overall death rate), and 1−*β* is power. This formula shows the relationship between hazard ratio (or effect size) *Δ*, variability *p*(1−*p*), and statistical power given all the other parameters being fixed. Clearly, to detect a specific effect *Δ* between mutations and non-mutations, the power can be too low when there is little variability in a marker. This suggests that we can compute a lower and upper bound of the mutation rate from (1), so that there is sufficient power (⩾80%) to detect a specific effect, *Δ*, if the mutation rate falls between the bounds. When marker data from *n* patients (*n*⩽*N*) are available, but survival data are not, we can construct a 95% confidence interval for mutation rate and compare it with the bounds. If the 95% confidence interval falls completely below the lower bound (or completely above the upper bound), it suggests that the marker might have too little variability to be effective in predicting survival, even if marker data were collected from all *N* patients. In such circumstances, investigators can make informative decisions regarding whether they want to continue data collection on a marker that is unlikely effective, or direct resources to other markers showing more promise.

To decide how big *n* should be, we provide the following formula: 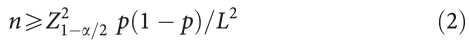 where *p* can be taken as 0.5 and *L* is a prespecified precision defined as the width of a 95% confidence interval. By using a small *L*, we can expect to have an accurate estimate for mutation rate based on only *n* (*n*⩽*N*) data points. It is important to note that, in addition to variability, effect size plays an important role in (1). When calculating the lower and upper bound (or simply the variance bound) at 80% power, we have to introduce a value for effect size. Unfortunately, the true effect size of a marker is unknown and cannot be estimated in the absence of survival data. Under such circumstances, supplying a value lower than the true effect size results in a higher variance bound, making it easier to reject a marker; supplying a value larger than the true effect size would only make it harder to reject a marker. We recommend supplying an upper bound for effect size to minimise the chance of throwing away important markers that may have very low variability, but huge effects on survival.

### Continuous protein marker data

The protein markers under investigation were assessed by immunohistochemistry. The scoring system was based upon the proportion of cells that were stained and the intensity of staining (Hoos and Cordon-Cardo, 2001). The final score took continuous values between 0 and 5. Similar to the mutation data, our goal was to develop a stopping rule for protein marker data, which might be applied after the first *n* (*n*⩽*N*) patients have been observed. If the variance of a protein marker is very small, it will likely have little prognostic value. The method illustrated here is again useful in the early stages of a study, when survival outcomes are not yet available. Assuming the Cox proportional hazards model, Hsieh and Lavori (2000) derived a sample size formula in which the variance of a continuous variable *σ*^2^ satisfies 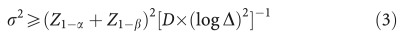 where *Δ* is the hazard ratio associated with one unit of increase in marker values. Similar to the binary marker case, a lower bound for marker variance can be computed by solving (3) for *σ*^2^, such that there is at least 80% power to detect a specific survival effect *Δ*, given the overall death rate *d* (*D*=N^*^d). Unlike in the binary marker case, there is no upper bound for marker variance in the continuous case. Again, effect size plays an important role in (3), in addition to variability. We do not want to underestimate the true effect size of a marker when calculating the variance lower bound. On the other hand, overestimating the true effect size would only make the method conservative.

If only continuous markers were evaluated in a study, one could use the following formula to compute the required sample size *n* to satisfy a certain precision *L*: 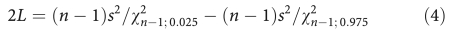 where *s*^2^ is an estimate of *σ*^2^, according to a pilot study. However, when both binary and continuous markers are evaluated in a study, there is no need to compute *n* twice. In that case, one can compute *n* based on formula (2) because of its simplicity.

## Results

### Binary mutation marker data

**Figure 1:**
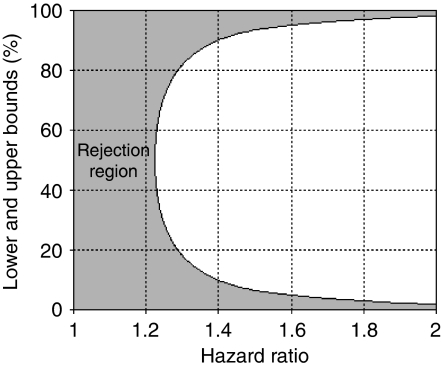
Lower and upper bounds calculated at 80% power, *α*=0.05, overall death rate *d*=0.6, and total sample size *N*=1000 for comparison with mutation rate estimated from *n* (*n*⩽*N*) patients. The shaded area represents the rejection region. A 95% confidence interval of mutation rate from *n* patients falling completely within this region suggests that the marker has little variability and likely insufficient power to predict survival, even if all data from *N* patients were collected.

We need at most *n*=97 (=1.96^2^ × 0.25/0.1^2^) patients to satisfy a 0.1 precision in evaluating a binary mutation marker (in formula (2), let *p*=0.5, as it gives the highest possible value for the right hand side). Table 1 displays the lower and upper bound of mutation rate, denoted as pL and pU, for a range of overall death rates and effect sizes where power is fixed at 80%, *N*=1000 and *α*=0.05. The bounds add up to 1 for each combination of overall death rate *d* and effect size *Δ* because of the symmetry in the left side of formula (1). When the overall death rate is lower than 20% and the effect size is also low, there are no solutions for pL and pU, because the power is insufficient (⩽80%) regardless of the mutation rate. Figure 1 displays pL and pU, when power is fixed at 80%, *α*=0.05, *d*=0.6, and *N*=1000. If the 95% confidence interval for mutation rate of a genetic marker falls completely in the grey area, it suggests little variability and effectiveness in the marker. For our study, we evaluated mutation markers, such as PI3 kinase, K-ras, B-raf, TGF*β*R-II, and MSI (Table 2). At the time of the development of this method, we had collected data for more than 97 patients. Table 2 displays the results based on all the data available at that time. We thought it reasonable to expect a 0.6 overall death rate among the *N*=1000 registered patients, and a hazard ratio of no more than 1.5 (i.e., 1.5 was an upper bound for the effect size between the mutation and non-mutation groups). As shown in Table 1, the lower and upper bound of mutation rate are 0.067 and 0.933, respectively (for a 0.6 overall death rate and 1.5 hazard ratio). Among the markers, only TGF*β*R-II had a 95% confidence interval of 0.019 and 0.057, falling completely below the 0.067 lower bound. This reveals that less than 6% of the population had TGF*β*R-II mutations, a range unlikely to have sufficient power (>80%) to predict prognosis, even if we gathered TGF*β*R-II mutation data from all *N*=1000 patients. Thus, we decided to stop further genetic analysis on TGF*β*R-II, and focus attention on the other markers.

### Continuous protein marker data

**Figure 2:**
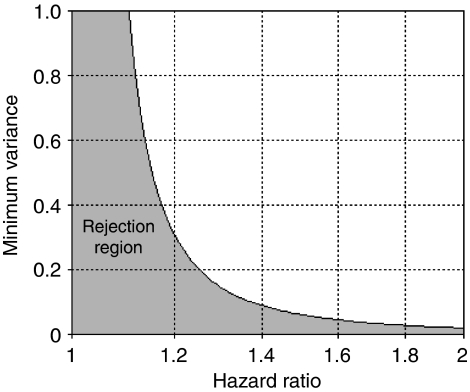
Minimum variance calculated at 80% power, *α*=0.05, overall death rate *d*=0.6, and total sample size *N*=1000 for comparison with marker variance estimated from *n* (*n*⩽*N*) patients. The shaded area represents the rejection region. A 95% confidence interval of marker variance from *n* patients falling completely within this region suggests that the marker has little variability and likely insufficient power to predict survival, even if all data from *N* patients were collected.

Table 3 presents the minimum variance required to detect a specific hazard ratio for a range of overall death rate values when power is fixed at 80%, *N*=1000, and *α*=0.05. In our study, protein markers were measured for Bcl2, cyclin D1, E-cadherin, hMLH1, Ki67, MDM2, and P53. We computed a 95% confidence interval for the variance of each marker (Table 4). Again, the overall death rate was expected to be 0.6, and the true effect size was not more than 1.5 for one unit of increase in the protein marker values. According to Table 3, the minimum variance required for each marker is 0.063. As the lower confidence limits of all the markers were larger than 0.063, none of the markers met the stopping criterion at this early stage of the study. Figure 2 displays the rejection region of variance when power is fixed at 80%, *α*=0.05, *d*=0.6, and *N*=1000, which is the case for our study.

## Discussion

The prospect that we might use the molecular characteristics of tumours to determine patient prognosis and predict response to chemotherapy is compelling. Studies to date have shown promising results, and there is every expectation that continued research will further improve our prognostic and predictive abilities. Although it is tempting to perform molecular analyses on an entire study sample, depending on the size of the study sample and the variability in a marker, the analysis might not be informative. In this study, we have illustrated one potential approach to evaluate marker effectiveness in the early stage of a study when survival data are not available, and the number of markers under consideration was limited. We recommend supplying an upper bound for the true effect size when calculating the marker variance bounds. In doing so, we minimise the chance of throwing away important markers that may have very low variability but huge effects on survival. The method is conservative; in that we do not abandon markers early unless markers show extremely low variability. However, if any markers are identified ineffective, the savings in money, time, and resources may be significant.

Institutional review boards and funding agencies generally demand power calculations (Friedman *et al*, 1999) as a requisite for study approval. The stakes are lower for molecular studies upon existing samples, but the ethical impetus remains to make efficient use of resources and precious, often irreplaceable, patient samples. Our approach helps identify uninformative markers in the early stage of a large molecular study to conserve time and resources. To fully assess this approach, future researchers should consider evaluating the real gain and loss of applying this approach on a large and completed study.

## Figures and Tables

**Table 1:** Lower (pL) and upper (pU) bounds calculated at 80% power, *α*=0.05, and total sample size *N*=1000

	**Hazard ratio *Δ***															
	**1.5**		**2**		**2.5**		**3**		**3.5**		**4**		**4.5**		**5**	
**Overall death rate *d***	**pL**	**pU**	**pL**	**pU**	**pL**	**pU**	**pL**	**pU**	**pL**	**pU**	**pL**	**pU**	**pL**	**pU**	**pL**	**pU**
0.05	NA	NA	NA	NA	17.9	82.1	11.6	88.4	8.6	91.4	6.9	93.1	5.8	94.2	5	95
0.1	NA	NA	15.2	84.8	8	92	5.4	94.6	4.1	95.9	3.3	96.7	2.8	97.2	2.4	97.6
0.15	NA	NA	9.5	90.5	5.2	94.8	3.5	96.5	2.7	97.3	2.2	97.8	1.9	98.1	1.6	98.4
0.2	25.1	74.9	6.9	93.1	3.8	96.2	2.6	97.4	2	98	1.6	98.4	1.4	98.6	1.2	98.8
0.25	18.4	81.6	5.4	94.6	3	97	2.1	97.9	1.6	98.4	1.3	98.7	1.1	98.9	1	99
0.3	14.7	85.3	4.5	95.5	2.5	97.5	1.7	98.3	1.3	98.7	1.1	98.9	0.9	99.1	0.8	99.2
0.35	12.2	87.8	3.8	96.2	2.2	97.8	1.5	98.5	1.1	98.9	0.9	99.1	0.8	99.2	0.7	99.3
0.4	10.5	89.5	3.3	96.7	1.9	98.1	1.3	98.7	1	99	0.8	99.2	0.7	99.3	0.6	99.4
0.45	9.2	90.8	2.9	97.1	1.7	98.3	1.2	98.8	0.9	99.1	0.7	99.3	0.6	99.4	0.5	99.5
0.5	8.2	91.8	2.6	97.4	1.5	98.5	1	99	0.8	99.2	0.6	99.4	0.5	99.5	0.5	99.5
0.55	7.4	92.6	2.4	97.6	1.4	98.6	0.9	99.1	0.7	99.3	0.6	99.4	0.5	99.5	0.4	99.6
0.6	6.7	93.3	2.2	97.8	1.2	98.8	0.9	99.1	0.7	99.3	0.5	99.5	0.5	99.5	0.4	99.6
0.65	6.2	93.8	2	98	1.1	98.9	0.8	99.2	0.6	99.4	0.5	99.5	0.4	99.6	0.4	99.6
0.7	5.7	94.3	1.9	98.1	1.1	98.9	0.7	99.3	0.6	99.4	0.5	99.5	0.4	99.6	0.3	99.7
0.75	5.3	94.7	1.7	98.3	1	99	0.7	99.3	0.5	99.5	0.4	99.6	0.4	99.6	0.3	99.7
0.8	4.9	95.1	1.6	98.4	0.9	99.1	0.6	99.4	0.5	99.5	0.4	99.6	0.3	99.7	0.3	99.7
0.85	4.6	95.4	1.5	98.5	0.9	99.1	0.6	99.4	0.5	99.5	0.4	99.6	0.3	99.7	0.3	99.7
0.9	4.4	95.6	1.5	98.5	0.8	99.2	0.6	99.4	0.4	99.6	0.4	99.6	0.3	99.7	0.3	99.7
0.95	4.1	95.9	1.4	98.6	0.8	99.2	0.5	99.5	0.4	99.6	0.3	99.7	0.3	99.7	0.3	99.7

NA=there is no solution for mutation rate.

**Table 2:** Mutation rate and 95% confidence limits estimated from binary marker data

**Mutation marker**	***n***	**Mutation rate estimated from *n* patients (95% confidence interval)**	**Total sample size**	**Overall death rate**	**Upper bound of effect size**	**Lower and upper bounds for mutation rate**	**Stopping**
PI3 kinase	131	0.15 (0.09, 0.21)	1000	0.6	1.5	(0.067, 0.933)	No
K-ras	223	0.48 (0.41, 0.55)	1000	0.6	1.5	(0.067, 0.933)	No
B-raf	204	0.30 (0.24, 0.37)	1000	0.6	1.5	(0.067, 0.933)	No
TGF*β*R-II	393	0.038 (0.019, 0.057)	1000	0.6	1.5	(0.067, 0.933)	Yes
MSI	446	0.24 (0.20, 0.28)	1000	0.6	1.5	(0.067, 0.933)	No

MSI=microsatellite instability.

**Table 3:** Minimum variance calculated at 80% power, *α*=0.05, and total sample size *N*=1000

	**Hazard ratio *Δ***							
**Overall death rate *d***	**1.5**	**2**	**2.5**	**3**	**3.5**	**4**	**4.5**	**5**
0.05	0.752	0.257	0.147	0.102	0.079	0.064	0.055	0.048
0.1	0.376	0.129	0.074	0.051	0.039	0.032	0.027	0.024
0.15	0.251	0.086	0.049	0.034	0.026	0.021	0.018	0.016
0.2	0.188	0.064	0.037	0.026	0.020	0.016	0.014	0.012
0.25	0.150	0.051	0.029	0.020	0.016	0.013	0.011	0.010
0.3	0.125	0.043	0.025	0.017	0.013	0.011	0.009	0.008
0.35	0.107	0.037	0.021	0.015	0.011	0.009	0.008	0.007
0.4	0.094	0.032	0.018	0.013	0.010	0.008	0.007	0.006
0.45	0.084	0.029	0.016	0.011	0.009	0.007	0.006	0.005
0.5	0.075	0.026	0.015	0.010	0.008	0.006	0.005	0.005
0.55	0.068	0.023	0.013	0.009	0.007	0.006	0.005	0.004
0.6	0.063	0.021	0.012	0.009	0.007	0.005	0.005	0.004
0.65	0.058	0.020	0.011	0.008	0.006	0.005	0.004	0.004
0.7	0.054	0.018	0.011	0.007	0.006	0.005	0.004	0.003
0.75	0.050	0.017	0.010	0.007	0.005	0.004	0.004	0.003
0.8	0.047	0.016	0.009	0.006	0.005	0.004	0.003	0.003
0.85	0.044	0.015	0.009	0.006	0.005	0.004	0.003	0.003
0.9	0.042	0.014	0.008	0.006	0.004	0.004	0.003	0.003
0.95	0.040	0.014	0.008	0.005	0.004	0.003	0.003	0.003

**Table 4:** Variance and 95% confidence limits estimated from continuous protein marker data

**Protein marker**	***n***	**Marker variance estimated from *n* patients (95% confidence interval)**	**Total sample size**	**Overall death rate**	**Upper bound of effect size**	**Lower bound for marker variance**	**Stopping**
Bcl2	156	0.64 (0.52, 0.82)	1000	0.6	1.5	0.063	No
CyclinD1	124	0.5 (0.40, 0.65)	1000	0.6	1.5	0.063	No
E-cadherin	174	0.64 (0.53, 0.81)	1000	0.6	1.5	0.063	No
hMLH1	93	1.06 (0.81, 1.44)	1000	0.6	1.5	0.063	No
Ki67	92	0.64 (0.49, 0.88)	1000	0.6	1.5	0.063	No
Mdm2	179	0.22 (0.18, 0.28)	1000	0.6	1.5	0.063	No
P53	174	2.88 (2.36, 3.60)	1000	0.6	1.5	0.063	No
